# Estimating the costs of supporting safety-net transformation into patient-centered medical homes in post-Katrina New Orleans

**DOI:** 10.1097/MD.0000000000004990

**Published:** 2016-09-30

**Authors:** Hui Shao, Lisanne Brown, Mark L. Diana, Laura A. Schmidt, Karen Mason, Carlos Irwin Oronce, Lizheng Shi

**Affiliations:** a School of Public Health and Tropical Medicine, Tulane University; b Louisiana Public Health Institute, New Orleans, LA; c University of California, San Francisco, CA.

**Keywords:** cost evaluation, PCMH clinics, safety-net clinics

## Abstract

There is a need to understand the costs associated with supporting, implementing, and maintaining the system redesign of small and medium-sized safety-net clinics. The authors aimed to understand the characteristics of clinics that transformed into patient-centered medical homes and the incremental cost for transformation.

The sample was 74 clinics in Greater New Orleans that received funds from the Primary Care Access and Stabilization Grant program between 2007 and 2010 to support their transformation. The study period was divided into baseline (September 21, 2007–March 21, 2008), transformation (March 22, 2008–March 21, 2009), and maintenance (March 22, 2009–September 20, 2010) periods, and data were collected at 6-month intervals. Baseline characteristics for the clinics that transformed were compared to those that did not. Fixed-effect models were conducted for cost estimation, controlling for baseline differences, using propensity score weights.

Half of the 74 primary care clinics achieved transformation by the end of the study period. The clinics that transformed had higher total cost, more clinic visits, and a larger female patient proportion at baseline. The estimated incremental cost for clinics that underwent transformation was $37.61 per visit per 6 months, and overall it cost $24.86 per visit per 6 months in grant funds to support a clinic's transformation.

Larger-sized clinics and those with a higher female proportion were more likely to transform. The Primary Care Access and Stabilization Grant program provided approximately $24.86 per visit over the 2 and 1/2 years. This estimated incremental cost could be used to guide policy recommendations to support primary care transformation in the United States.

## 1 Introduction

The patient-centered medical home (PCMH) has been proposed as a promising model for transforming primary care.^[1–3]^ In general, this model can be described as a physician-led team-based approach to primary care, in which providers across multiple sites are connected under the aim of increasing the access (eg, expanding practice hours), quality, and continuity of care (eg, constructing new channels of communication between providers and patients across the spectrum of care).^[4]^ The Patient Protection and Affordable Care Act (ACA) adopted the concept of the PCMH as a model for service delivery system reform. It states in section 3502 that health systems should “develop and implement interdisciplinary, interprofessional care plans that integrate clinical and community preventive and health promotion services for patients.”^[5]^ Facilities that aim to transform into PCMHs should go through a PCMH accreditation process for PCMH recognition. The most widely used PCMH recognition process has been designed by National Committee on Quality Assurance (NCQA). Through an extensive application and site visit process, primary care practices are evaluated by the NCQA on 6 key elements: access during office hours, use of data for patient population management, care management supporting the self-care process for patients, referral tracking and follow-up, and implementation of continuous quality improvement.^[6,7]^ Those that have attained these characteristics are awarded “NCQA recognition” as PCMHs.^[8]^

Current health services research is aimed at understanding and evaluating the efficacy of the PCMH model, especially for its impact on cost, patient satisfaction, and quality improvement. A great deal of the literature has been conducted during the past decade.^[6,9–16]^ To date, much of this research has focused on improving quality outcomes in large integrated delivery systems. However, there is a small, but growing literature on the challenges of establishing PCMHs in the safety net, including a series of prior studies on the post-Katrina New Orleans experience.^[17–19]^ There is an urgent need to understand the costs associated with supporting, implementing, and maintaining the system redesign of small and medium-sized primary care practices.^[20]^ Small practices account for the lion's share of US primary care delivery, serving 20 million Americans, including some of the most vulnerable populations in rural communities, and also those covered by Medicaid and the uninsured—commonly referred to as the “safety-net population.”^[21–23]^

Previous studies have found that the economic costs of transforming primary care practices into medical homes can be significant.^[24]^ Over time, however, the PCMH transformation process seems to pay off by sustaining lower costs of delivering care.^[25]^ Raising funds to undergo the initial process of practice transformation is especially challenging for those small and medium-sized clinics without external funding.^[26]^ Federal funding for PCMH demonstration projects has helped to promote diffusion of the PCMH model nationally.^[27]^

In New Orleans after Hurricane Katrina, safety-net clinics throughout affected areas embarked on a system-wide effort to rebuild and transform the healthcare safety net using the PCMH model. Their efforts were supported by a $100-million federal grant: the Primary Care Access and Stabilization Grant (PCASG).^[18,28–30]^ The PCASG program established a framework for rapidly and efficiently expanding primary healthcare access to residents in the 4-parish Greater New Orleans area, which was severely impacted by Hurricane Katrina. The PCASG Notice of Award (NOA) required participating organizations to “establish a quality assurance or quality improvement program as part of daily operations,” and implemented quality standards consistent with the PCMH as the reimbursement reference. Practices that successfully gained NCQA recognition were given special financial bonuses. A New Orleans based, nonprofit organization—the Louisiana Public Health Institute (LPHI)—administered the PCASG funds. LPHI adopted the *NCQA Physician Practice Connection Patient-Centered Medical Home* (2008) as a guidance tool for the PCMH-linked changes.^[31]^ Minimum standards for quality and access had been established for all PCASG clinics by June 2008 (eg, 24/7 phone response, evidence-based guidelines, and specialty referral arrangements). A voluntary Quality Improvement Incentive Payment Program was also established to offer additional financial resources for clinics recognized by NCQA as PCMHs.

The objective of this study was to understand the characteristics of safety-net clinics that transformed into PCMHs under the PCASG grant and the incremental cost for this transformation process in Greater New Orleans. Our strategy was to compare the differences in PCASG grant expenditures and clinic characteristics for those clinics that successfully transformed into PCMHs and those that did not. Since PCASG is the only major financing source to support those safety-net clinics to transform into PCASG, by tracking the expenditure of the fund provided us with a valid measurement to the cost for PCMH transformation. In our study, the grant expenditure was compared using difference-in-difference methods to estimate the actual incremental cost on supporting PCMH transformation.

We also examined the associations between clinic-level characteristics and cost measures in primary care practices under the PCASG program. This allowed us to identify characteristics of clinics that were more efficient in scaling up into medical homes.

## 2 Methods

### 2.1 Data sources

Administrative monitoring data, collected by the Louisiana Public Health Institute, provided systematic data over time, capturing changes in New Orleans practices, affording us with empirical data to evaluate the incremental costs of PCMH transformation of safety-net clinics. Specifically, we used the PCASG program data over a 3-year observation period (September 21, 2007–September 20, 2010), including the patient encounter registry, the services delivery register, grant expenditure data, and the NCQA recognition profiles of clinics. The observation period started shortly after the PCASG program began, and after all clinics enrolled over the entirety of PCASG. A total of 110 primary care clinics, which were nested within 24 larger grantee organizations, comprised the sample followed for this study. The grant expenditure data were collected at 6-month intervals, producing 6 waves of data over the 3-year observation period. We also tracked if the PCASG clinics became PCMHs based on whether or not they received NCQA recognition. This provided opportunities to study differences between primary care practices that became medical homes and those that did not.

### 2.2 Sample selection

All clinics in the PCASG network were eligible for payment incentives if they were successful in achieving NCQA status as PCMHs. LPHI scaled bonus payments to reflect the number of points NCQA awarded each clinic. Three rounds of incentive payments occurred during the grant period, with clinic payments ranging from $55,826 to $135,053. Prior analyses suggested that LPHI's payment incentives created a strong incentive for clinics within the PCASG program to seek NCQA recognition as medical homes.^[18]^

**Figure 1:**
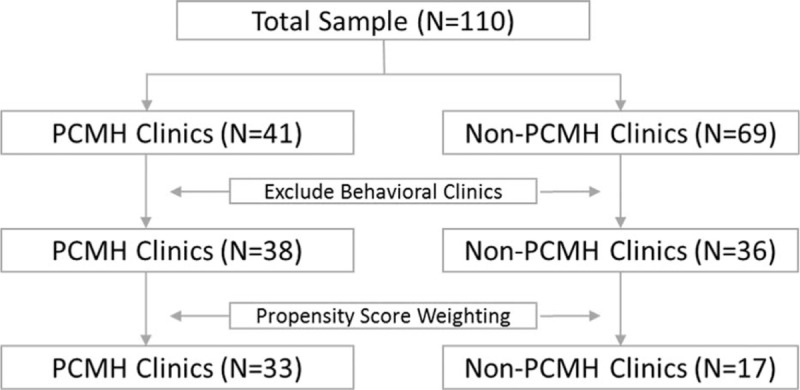
Flow chart for sample selection.

All of the PCASG clinics were encouraged to apply for NCQA PCMH certification as part of the quality incentive program. Many behavioral health clinics, however, decided not to apply, as they believed that the certification was geared more towards primary care clinics. Several of the elements required for certification, particularly around electronic medical record (EMR) adoption, were difficult to implement in the behavioral health setting. Although behavioral health clinics occupied a large proportion of safety-net clinics, we excluded them from our total sample and conducted the analysis on primary care clinics exclusively (Fig. 1).

Furthermore, baseline characteristics of PCMHs and non-PCMHs differed in many aspects that likely confound our outcomes of interest (eg, clinic size, sex proportion, and race proportion). For example, larger clinics may tend to be more likely to transform into PCMHs due to greater organizational capacity.^[32]^ Considering larger clinics are different from smaller clinics in many ways and might not be comparable, we used a propensity score weighting (PSW) technique to reconstruct 2 comparable groups at the baseline wave. Because our study sample is small, the PSW technique is appropriate since it retains most of the sample.^[33]^ The variables used to fit our propensity score model included total visits, mean age, percent of African American patients, percent of females, total full-time equivalent (FTE) physicians, and percent of uninsured patients. Noting that common support is a critical but often neglected presumption in propensity score matching,^[34]^ we excluded clinics which were “off support,” retaining only 33 PCMH clinics and 17 non-PCMH clinics. After calculating each clinic's propensity score, weights were calculated and applied. Each PCMH clinic was given a weight of 1, and the non-PCMH clinics were assigned weights equal to their propensity score/(1 − propensity score). Detailed methodology can be found in the study by Austin.^[35]^

### 2.3 Measures

We estimated costs by tracking actual PCASG program expenditures to each clinic in the following categories: personnel, fringe benefits, travel, equipment, supplies, contracts, and alteration and renovation.

The PCMH status was defined on the basis of NCQA recognition as a PCMH. PCMH transformation in New Orleans was a long-term process.^[24]^ During the time leading up to a clinic's application for NCQA recognition, we expected to observe a sharp increase in expenditures, reflecting the clinic's investment in upgrading the accessibility, quality, and continuity of its care. We therefore coded the data to identify the 6-month wave of data collection before the NCQA application period along with the period concurrent with the application. Based on the organization's PCMH transformation status, the costs were then summarized in terms of baseline practice expense, incremental cost of PCMH transformation, and maintenance of practice change (waves after transformation). Since all the PCMH recognitions were made in the middle of wave 3, we defined the general transformation period as waves 2 to 3, with wave 1 defined as the baseline period.

### 2.4 Statistical analysis and model specification

Baseline characteristics included total outpatient visits, FTE physicians, mean age of patients, the proportion of African American, the proportion of females, percent of uninsured patients, and surplus grant. (The surplus grant for the current wave was measured by surplus grant funds of the previous wave plus the newly allocated grant funds for the current wave, minus the grant expenditure of the previous wave.) Descriptive analyses compared non-PCMH clinics with those that became PCMHs over the course of the observation period using the Mann–Whitney test or chi-square test, as appropriate. We calculated the mean age of the patients, the proportion of patients that were African American, the proportion of females, and the proportion of uninsured patients for each clinic for each wave from the encounter data.

### 2.5 Fixed and random-effects modeling for cost measures

Given the panel structure of our data, we used 2 different methods to cope with different assumptions. A fixed-effects model at clinic level was better suited to the situation where endogeneity posed a threat to our model estimation, whereas a random-effects model was more appropriate when endogeneity was not a concern for estimating the incremental cost of PCMH transformation.^[36]^ After fitting both fixed-effects and random-effects models, we conducted a *Hausman* test to identify which model was more appropriate to estimate the incremental cost of PCMH transformation.

Because costs vary with the size of a clinic, which can be measured by the number of FTE physicians in the practice and the number of total outpatient visits. However, the FTE physicians in the data set only recorded contracted FTE physicians; thus both total cost and total cost per visit were used as study outcomes. We constructed separate models for both outcome variables. Explanatory variables included total visits, FTE physicians, mean age of patients, the proportion of African American patients, the proportion of female patients, the percent of uninsured patients, and surplus grant. All of the above variables were potential cost drivers based on the literature. Our independent variable of interest is PCMH status. Although a PCMH clinic may have achieved 3 possible NCQA levels of recognition,^[1–3]^ because of our small sample size and because previous studies did not find a significant association between higher expenditures and NCQA recognition levels,^[37]^ we aggregated those levels and constructed a binary, time-variant indicator for clinics’ PCMH status.

We ran our fixed-effects model on the first 3 waves of data (transformation-period model) to capture the exact incremental cost of transforming into PCMH clinics, given our identification for the second and third waves being the transformation period. We also ran the model on all of the 6 waves (overall-period model) to evaluate the amount of funding needed to support a PCMH clinic in the whole project period. All the regressions were weighted by propensity score weights, and errors were clustered at organization level to cope with potential heteroscedasticity. All the analyses were performed by STATA 12.0. Details regarding the application of weighted regression by PSW can be found in the study by Hirano and Imbens.^[38]^

### 2.6 Sensitivity analysis

To strengthen the validity of the study, we conducted a sensitivity analysis to test if our findings were robust to different model specification and different study samples. The explanatory variables included in the model were identical to the previous 2 models; however, the main independent variable of interest was different. Instead of creating a binary, time-variant indicator for PCMH status, we constructed a dummy set for period identification. A clinic could be in one of the baseline (wave 1), transformation (wave 2–3), or maintenance (wave 4–6) period. This alternative modeling strategy was applied on a different samples compared with the previous 2 models. Because about one-fourth of the clinics closed during the maintenance period, only clinics that survived the whole 6 waves were included in the sensitivity analysis to insure a balanced panel. The results from our sensitivity analyses were expected to be similar to the results of the transformation-period model.

This is a retrospective study which used data at clinical level, thus no patients’ informed consent was involved. This study has been approved by Tulane University Biomedical Institutional Review Board.

## 3 Results

### 3.1 Baseline characteristics of primary care clinics

Table 1 presents the baseline characteristics of PCMH primary care clinics. The mean total cost of PCMH clinics was more than double the mean of non-PCMH clinics, although this difference is not statistically significant. Clinics that would transform into PCMHs had a mean of 3197 total outpatient visits, roughly 3 times the mean total visits of non-PCMH clinics (1040) at baseline (*P* = 0.004). PCMH clinics had 2.22 contracted FTE physicians at the baseline, whereas non-PCMHs had approximately 1 contracted FTE physician. PCMH transformation was also correlated with a higher percent of female patients (*P* = 0.033). Clinics that transformed into PCMHs had approximately 60% female patients, which was significantly more than non-PCMH clinics.

**Table 1:**
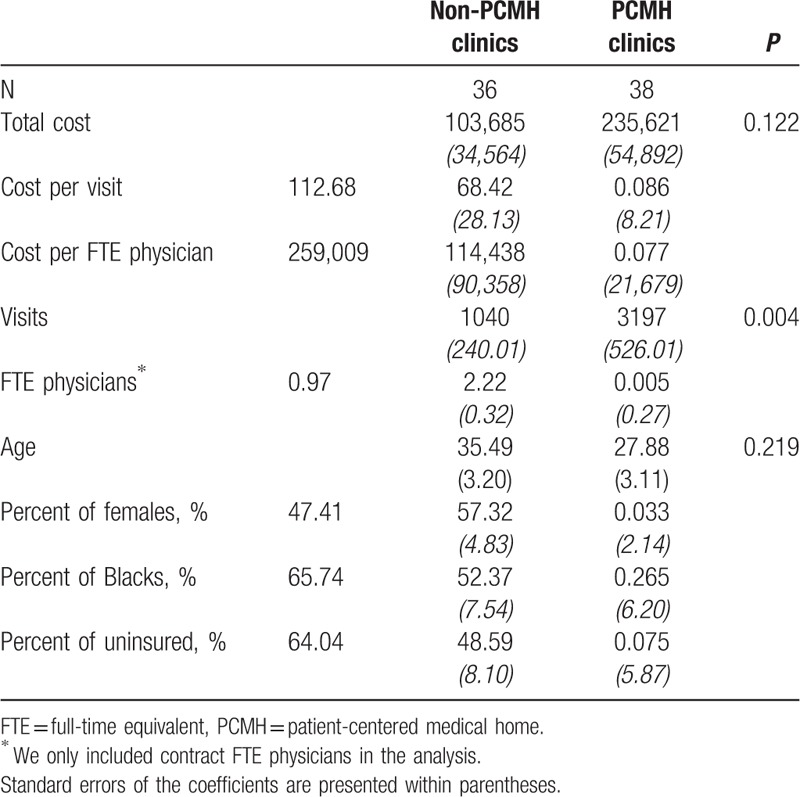
Baseline (wave 1) characteristics of PCMH and non-PCMH primary care clinics.

In addition, we found that the percent of uninsured patients was marginally correlated with PCMH transformation (*P* = 0.075). Table 2 presents the results of a balance test after PSW. All of the baseline characteristics were comparable (all *P* values >0.05).

**Table 2:**
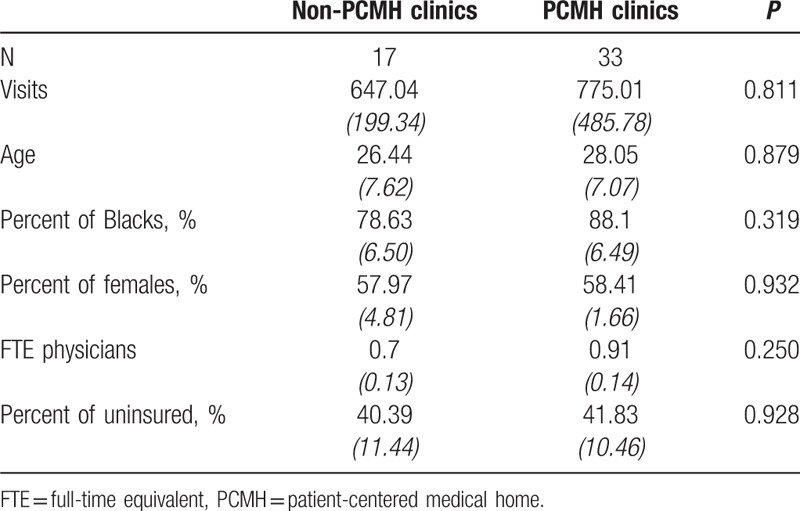
Baseline characteristics in primary care clinics after propensity score weighting on common support.

### 3.2 Econometric modeling

The *Hausman* test suggested that both the cost model and cost per visit model suffered from endogeneity, and that the fixed-effects model was more appropriate than random-effects model in this situation (*P* < 0.05). Therefore, we applied the fixed-effects model to the PSW sample. The results are presented in Table 3 for the total cost model and Table 4 for the cost per visit model, respectively.

**Table 3:**
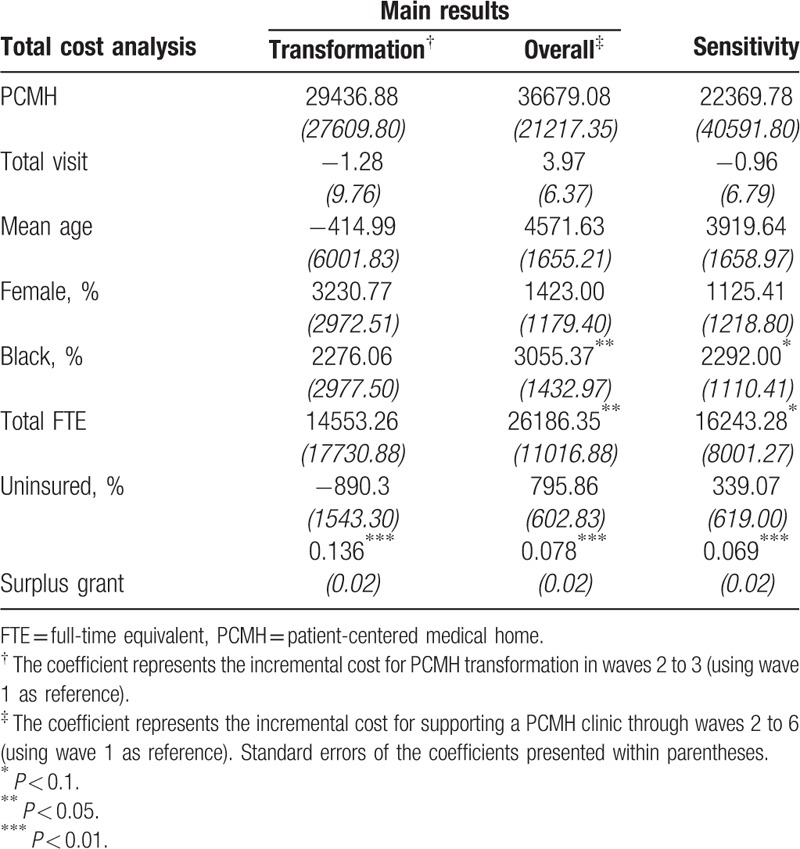
Regression model of PCMH transformation on total cost.

**Table 4:**
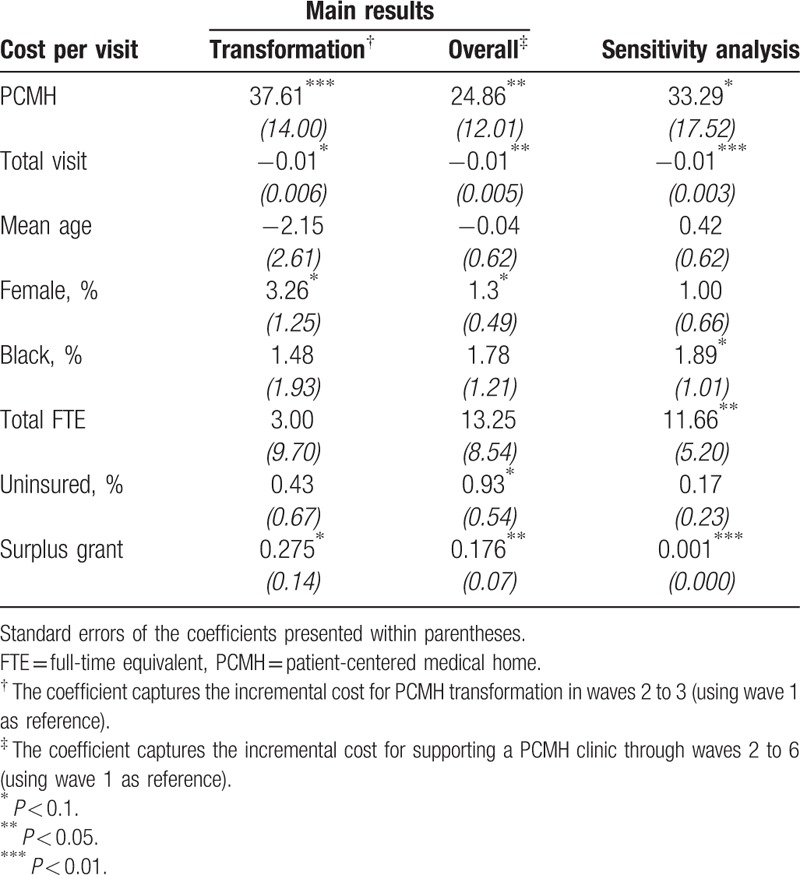
Regression model of PCMH transformation on cost per visit.

The transformation-period model provided an estimated value for the incremental cost of about $29,439 per wave for PCMH transformation, although the difference was not statistically significant (Table 3; *P* = 0.439). Our overall-period analysis indicated that PCMH clinics spent $36,679 more per wave from PCASG. Since we defined wave 1 as the baseline wave, the total amount of incremental costs that the PCASG grant provided to help clinics complete the PCMH transformation amounted to $183,395.

Table 4 presents the main models and sensitivity analysis results of our cost-per-visit model. The estimates for incremental cost per visit were similar between the transformation-period model and the sensitivity analysis. Compared with non-PCMH clinics, PCMH clinics cost $37.61 more per visit in the transformation period (*P* < 0.01). Since we had identified the transformation period as 1 year (2 waves), we could approximate the clinic's incremental cost by simply multiplying $37.61 by the number of yearly total outpatient visits in that clinic. We also found that 1 visit increase was associated with a decrease in cost by $0.01 dollars per visit (*P* < 0.01). In addition, a 1% increase in female patients was associated with an increase in cost by $3.26 per visit (*P* < 0.1). On average, it cost the PCASG grant $24.86 more per patient visit to motivate a safety-net clinic to transform into a PCMH clinic (*P* < 0.05) during a two and a half year interval.

## 4 Discussion

The purpose of this study was to understand the characteristics of safety-net clinics that transformed into PCMHs under the PCASG grant and the incremental cost for this transformation process in the Greater New Orleans area. Our results suggest that clinics that transform into PCMHs had certain advantageous characteristics, and hence, the most important of which is size, leading to the conclusion that PCMH transformation is an endogenous variable. Compared with the overall costs of non-PCMH clinics, those that underwent PCMH transformation had 3 times greater costs at baseline. In addition, a greater number of total outpatient visits were significantly associated with PCMH transformation. We also found that clinics with larger female patient proportion were more likely to transform into PCMH. The most probable reason is that larger clinics were equipped with more infrastructure to provide OB/GYN or maternal services. These findings suggested that there may be advantages for larger clinics for successful PCMH transformation, and even under PCASG environment, smaller-sized safety-net clinics still faced challenges to transform into PCMHs. This finding suggested that policymakers should consider providing a greater incentive for smaller clinics that would otherwise be less likely to choose PCMH transformation.

Also, our results strongly suggest that clinics self-select into PCMH transformation. Thus a direct comparison between the costs for PCMHs and non-PCMHs on the posttransformation period has to take in account the baseline differences. The existence of the baseline difference may be explained by our empirical data and classic economic “learning-by-doing” theory that more physician experience reduces costs per visit.^[39,40]^ Our findings improved over prior studies, which were based solely on posttransformation data or did not adjust for self-selection bias associated with PCMH transformation.

Our cost per visit estimate was consistent across analyses and samples. From the baseline cost per visit at $68.42, the safety-net clinics that transformed into PCMHs spent $37.61 more per visit than those that did not (1-year period). Overall, the PCASG program spent $24.86 more per visit for clinics that transformed into PCMHs over 3 years. Our findings of 36.33% increase in total cost per visit across two and half year period should inform future health policy that attempts to transform and sustain primary care clinics into PCMHs.

Safety-net clinics pose special challenges for cost estimation studies, because they typically serve a population with distinctive characteristics from the general population^[23,41,42]^ (eg, insurance coverage, income, and racial proportion). Because of their low percentage of insured patients, claims data cannot be applied to support our cost evaluation. In addition, most safety net clinics in the Greater New Orleans area do not possess sufficient infrastructure (eg, electronic health record systems, personnel resources), making it difficult to collect their health-related data. To cope with this challenge and to obtain variables to support our cost estimation, we applied certain approaches, including merging the grant expenditure data with other data sources (eg, the patient encounter registration data, the services delivery registration data, and NCQA profiles of clinics) and aggregating patient-level information up to clinic-level variables.

The panel structure of our data provides an advantage in coping with the endogeneity issue compared with previous studies that employed cross-sectional data.^[37,43,44]^ Furthermore, our study benefits from multiple methods, including matching techniques and econometric modeling. The PSW technique was used before applying econometric methods, which allowed us to preserve the “parallel trend” assumption.^[45]^ This assumption would have been violated if clinic characteristics or initial conditions were different as they would likely lead to divergent cost trajectories over time between PCMH and non-PCMH clinics. Thus, the combination of econometric methods with PSW to address biases presented by observables strengthened the validity of our findings.^[46,47]^

The small number of practices in our study limited our ability to examine the different incremental costs required for recognition as a level 1 or a level 3 PCMH. It is possible that it would cost less than $33.29 per visit to transform into a level 1 PCMH. However, a previous study found no evidence that the higher cost of PCMHs was associated with a higher level of NCQA recognition.^[37]^ In addition, the PSW process removed 19 non-PCMHs clinics (usually small-sized). Therefore, our finding may be less generalizable to the case mix of safety-net clinics.

Another limitation is the representativeness of the data on FTE physicians. Due to the dynamic nature of the safety-net clinics, we were only able to capture the officially contracted FTE physicians. The actual number of FTE physicians working in the clinics may be higher than the number we collected, which is the primary reason the cost per FTE physician was a secondary outcome.

In summary, our study found that the incremental cost of transformation to a PCMH amongst safety-net clinics in New Orleans was substantial and especially apparent at the per-visit level. To advance the model of the PCMH, policymakers need to provide adequate incentives that differentially target smaller clinics to help clinics overcome short-term cost barriers to transformation.
